# Whole genome sequence and LC-Mass for identifying antimicrobial metabolites of *Bacillus licheniformis* endophyte

**DOI:** 10.1186/s13568-024-01789-y

**Published:** 2024-12-20

**Authors:** Nourhan K. Soliman, Ahmad M. Abbas, Wafaa N. El tayeb, Mohammad Y. Alshahrani, Khaled M. Aboshanab

**Affiliations:** 1 Department of Microbiology and Immunology, Faculty of Pharmacy, Ain Shams University, Cairo, 11566 Egypt; 2 Department of Microbiology and Immunology, Faculty of Pharmacy, King Salman International University (KSIU), Ras Sudr, South Sinai Egypt; 3 Department of Microbiology, Faculty of Pharmacy, Misr International University (MIU), Cairo, 19648 Egypt; 4 Department of Clinical Laboratory Sciences, College of Applied Medical Sciences, King Khalid University, P.O. Box 61413, 9088 Abha, Saudi Arabia

**Keywords:** Virulence factor, Antibiotic resistance, Bacteriophage, *Enterococcus faceium*, *Enterococcus fecalis*, Stability

## Abstract

Antimicrobial resistance (AMR) represents a critical public health issue that requiring immediate action. Wild halophytic plants can be the solution for the AMR crisis because they harbor unique endophytes capable of producing potent antimicrobial metabolites. This study aimed at identifying promising and antimicrobial metabolites produced by endophytic/epiphytic bacteria recovered from the wild *Bassia scoparia* plant. Standard methods were employed for the isolation of endophytes/epiphytes. Whole genome sequence (WGS) using Oxford Nanopore technology followed by antiSMASH analysis coupled with advanced LC-MS spectroscopic analysis were used for identification of the active antimicrobial metabolites. This study identified *Bacillus licheniformis* strain CCASU-B18 as a promising endophytic bacterium from the *Bassia scoparia* plant. In addition, the strain showed broad-spectrum antibacterial activity against three standard and five MDR clinical Gram-positive and Gram-negative isolates, and antifungal activity against the standard *C. albicans* strain. Six main antimicrobial metabolites—thermoactinoamide A, bacillibactins, lichenysins, lichenicidins, fengycin, and bacillomycin—were verified to exist by whole genome sequencing for identifying the respective conserved biosynthetic gene clusters in conjunction with LC/MS-MS analysis. The complete genomic DNA (4125835) and associated plasmid (205548 bp) of the promising endophytic isolate were sequenced, assembled, annotated, and submitted into the NCBI GenBank database under the accession codes, CP157373. In conclusion, *Bacillus licheniformis* strain CCASU-B18, a promising endophytic bacterium exhibiting broad-spectrum antimicrobial activities, was isolated. Future research is highly recommended to optimize the culture conditions that will be employed to enhance the production of respective antimicrobial metabolites, as well as testing these compounds against a broader range of MDR-resistant pathogens.

**Supplementary Information:**

The online version contains supplementary material available at 10.1186/s13568-024-01789-y.

## Introduction

Antimicrobial resistance (AMR) is one of the most urgent public health concerns that needs to be dealt with rapidly. According to the World Health Organization (WHO), 1.27 million deaths were directly associated with antimicrobial drug resistance in 2019 (WHO 2023). The number of global deaths is predicted to reach 10 million by 2050, presumably being the primary cause of global deaths that year (Tang et al. 2023). Additionally, AMR poses a severe economic challenge; the World Bank, in a report titled “Drug-resistant infections: a threat to our economic future” stated that by 2030 1-3.4 trillion US dollars will be lost in terms of gross domestic product as a result of resistant infections (Miller-Petrie et al. 2017). One of the multiple ways to tackle this crisis is by means of innovating new antimicrobial therapeutics. Despite significant efforts by researchers, there remains a gap between newly developed antimicrobial agents and the rise of drug-resistant pathogens. There are still insufficient antibacterial drugs to combat WHO-priority pathogens that pose a threat to global health, which include carbapenem-resistant *K. pneumoniae*, *A. baumannii*, and *P. aeruginosa* (Miethke et al. 2021).

More than 60% of the newly innovated antibacterial compounds in the last 40 years are derived from nature (Miethke et al. 2021). Secondary metabolites derived from endophytic bacterial extracts are considered one of the natural sources of antibiotics (Martinez-Klimova et al. 2017). Endophytic bacteria, also known as “Endophytes” are a class of bacteria that are found within plant tissues. This type of bacteria is a mutualistic bacteria that live in symbiosis with the plant host without showing any signs of pathogenicity. Moreover, “Epiphytes” are another bacterial class related to plants that inhabit the plant’s surface (Afzal et al. 2019). Phenotypic investigation coupled with metagenomic/genomic analysis of the biosynthetic gene cluster of valuable secondary metabolites followed by spectroscopic analysis has been successfully used to screen and explore the nature of respective valuable metabolites (Eltokhy et al. 2021, 2022, 2024; Alam et al. 2022; Elbakary et al. 2024).

In recent years, Endophytes have attracted much attention due to their well-established secondary antimicrobial metabolites. Secondary antimicrobial metabolites isolated from endophytes include a range of chemical structures including peptides, alkaloids, steroids, terpenoids, polyketides, phenols, quinones, and flavonoids antibiotics (Yu et al. 2010; Martinez-Klimova et al. 2017). Endophytes still show immense potential to be a valuable source of newly discovered active metabolites (Gouda et al. 2016). This study aimed at identifying promising antimicrobial metabolites produced by the endophytic/epiphytic bacteria recovered from the wild *Bassia scoparia* plant, particularly those exhibiting broad-spectrum activities against standard and clinical bacterial and fungal pathogens.

## Materials and methods

### Collection of bacterial isolates

#### Plant collection and identification

A visibly healthy plant was collected using a shovel, sterile bag, and sterile gloves. It was collected from the First Settlement, New Cairo, Cairo, Egypt (30°03’26.7"N, 31°25’33.4"E) where it grew wildly in sandy, rocky soil. The plant was transported in an icebox to the lab where it was stored in the refrigerator for 24 h before processing. The plant was identified as *Bassia scoparia* using LeafSnap application (https://leafsnap.com/) (accessed on 01 September 2024) that acquired recognition accuracy of 94.38% as referred by previously reported (Turkoglu et al. 2021).

#### Plant processing

Plant parts, including roots, stems, and leaves were first rinsed with tap water, then cut into two parts using sterile scissors and cutters. The first part was just washing using sterile distilled water three times (each 1 min) to isolate epiphytes (War Nongkhla and Joshi 2014). The second part was subjected to surface sterilization to isolate endophytes. The surface sterilization process included immersion of plant parts in 70% ethanol for 1 min followed by treatment using 2.5% NaOCl (Sahu et al. 2022). Washing with sterile distilled water 3 times (each 2 min) was performed during the process and its end to avoid sterilant remnants of the plant tissue (Sahu et al. 2022).

### Isolation and purification of endophytes and epiphytes

The plant parts were cut using a sterile blade in a sterile glass petri dish. The plant parts were cut horizontally about 1 cm for a segment. The part subjected to surface sterilization was cut longitudinally to allow easier growth of endophytes. The segments were plated on nutrient agar, potato dextrose agar, and tap water-yeast extract agar, then, the plates were incubated for up to 4 weeks at 27 °C (Coombs and Franco 2003). During the incubation period, an examination of plates was conducted every three days to detect the presence of grown colonies, which were then purified by the streak plate method.

### Preliminary screening of antimicrobial activity

Screening of endophytic and epiphytic bacteria for antimicrobial activity was carried out by the perpendicular streak method (Ashitha et al. 2019). The tested bacteria were streaked vertically against standard and multi-drug resistant (MDR) pathogens including *E. coli* ATCC 25922, *S. aureus* ATCC 25923, *C. albicans* ATCC 14053, MDR *E. coli*, MDR *P. aeruginosa*, MDR *A. baumannii*, MDR *K. pneumoniae*, and MDR *K. terrigena*. The clinical MDR clinical isolates were obtained from the Culture Collection Ain Shams University (CCASU). The antimicrobial resistance pattern of clinical isolates used in this study is shown in Table 1. These strains were adjusted to a turbidity equivalent to 0.5 McFarland standard. The streak method was carried out on Mueller Hinton agar plates which were incubated for 24 h at 30 °C (Ashitha et al. 2019).

**Table 1:** Antimicrobial resistance pattern of tested MDR clinical isolates

Clinical isolate code	Resistance pattern*
MDR *E. coli*	TMP/SMX, AMC, CPD, CTX, CIP, GEN, MEM
MDR *P. aeruginosa*	MEM, CAZ, FEP, GEN, AMK, CIP
MDR *A. baumannii*	TMP/SMX, SAM, CTX, CRO, CIP, DOX, GEN, AMK, MEM
MDR *K. penumoniae*	MEM, TMP/SMX, AMC, CTX, CRO, CIP, DOX, GEN, AMK
MDR *Klebsiella terrigena*	MEM, AMC, SAM, FEP, CIP, DOX

*AMC = amoxicillin/clavulanic acid, AMK = Amikacin, CAZ = ceftazidime, CPD = cefpodoxime, CRO = ceftriaxone, CTX = cefotaxime, DOX = doxycycline, FEP = cefepime, CIP = ciprofloxacin, GEN = gentamicin, MEM = meropenem, SAM = ampicillin/sulbactam, TMP/SMX = trimethoprim-sulfamethoxazole.

### Preparation of the endophytic extract

The culture medium used in shake flask production is modified starch casein broth (Singh et al. 2021). The colonies of the selected isolates were cultured in 25 mL of broth for 24 h at 150 rpm and 30 °C. About 2 mL of the seed culture of optical density 0.7 was used to inoculate 22 × 50 mL Erlenmeyer flasks (each containing 100 mL of modified starch casein broth). The flasks were incubated for 72 h at 150 rpm and 30 °C (Singh et al. 2021). Bacterial mass separation was achieved through centrifugation at 12,000 rpm for 10 min followed by filtration through Whatman No.1 filter paper. The obtained filtrate was then mixed with ethyl acetate at a ratio of 1:1 (v/v). The extraction process was conducted twice to enhance the recovery of metabolite broth (Singh et al. 2021) and the collected organic layer was evaporated at 45 °C using a rotatory evaporator (IKA RV 10, China) to yield the crude extract (Eltokhy et al. 2021).

### Antimicrobial analysis of the collected extract

Antibacterial activity was evaluated by the agar well diffusion method (Eltokhy et al. 2021). The crude extract was dissolved in 1.5 mL of dimethyl sulfoxide (DMSO). The DMSO (diluted 60%) was used as a negative control. However, antifungal activity was tested by dissolving the extract in 60% diluted DMSO due to the high fungicidal effect of pure DMSO. The extract was tested against respective standard and clinical isolates which were adjusted to turbidity equivalent to 0.5 McFarland standard. Incubation was carried out at 37° C and plates were examined for zones of inhibition after 24 h.

### Genomics and antiSMASH analysis of the promising isolate

The chromosomal DNA was extracted using QIAamp^®^ DNA Mini Kit (cat. no. 51304, Qiagen, Hilden, Germany) using manufacturer protocol for Gram-positive bacteria, and the resulting DNA was quantified using Qubit 4 (Thermo Fisher Scientific, Massachusetts, USA). About ~ 400 ng of the collected chromosomal DNA was used for the library preparation using a Rapid Sequencing Kit (SQK-RAD004; Oxford Nanopore Technologies, Oxford, UK) according to manufacturer instructions. Sequencing was performed using MinION™ and R9.4.1 flow cells (FLO‐MIN106; Oxford Nanopore Technologies). The MinKNOW software version 23.11.5 (Oxford Nanopore Technologies) was used for data acquisition.

### Data analysis

MinION™ sequence reads (i.e., POD5 data) were converted into fastq files using Dorado basecall server ver. 7.3.9 (Oxford Nanopore Technologies) on AWS EC2 g4dn.xlarge. fastq files were classified using kraken2 (Wood and Salzberg 2014) and then visualized using recentrifuge (Martí 2019), then processed using Flye (Kolmogorov et al. 2019) reference-based assembly against the most abundant tax *Bacillus licheniformis* “taxid: 1402*”*. draft assembly was polished three times using medaka.

### Identification of the biosynthetic gene clusters

AntiSMASH version 7 (Antibiotics and Secondary Metabolite Analysis Shell) was used to align and analyze sequences for potential secondary metabolite gene clusters. (https://antismash.secondarymetabolites.org/#!/start) (Blin et al. 2021).

### LC-MS analysis of the obtained extract

The obtained extract was analyzed by LC-MS/MS using The Vanquish Core HPLC System coupled with a Thermo ScientificTM Q Exactive^™^ Hybrid Quadrupole-Orbitrap mass spectrometer equipped with an electrospray ionization (ESI) source coupled with an auto-sampler and surveyor UHPLC binary pump (Thermo Fisher Scientific, Bremen, Germany) and the process was carried out as previously described (Wong et al. 2020). The column used for separation was the Acquity C18 column (1.8 μm, 2.1 × 50 mm). The mobile phase used in the separation was LC-MS-grade water (solvent A) and methanol (solvent B), each consisting of 0.1% FA with a flow rate of 3 mL/min. Positive and Negative ion mode was done in full scan mass spectra acquisition from 200 to 3000 m*/z* with HCD collision energy of 20, 40, and 60%, as previously reported (Wong et al. 2020).

## Results

### Preliminary screening of antimicrobial activity

Preliminary screening results showed significant antimicrobial effects exhibited by both endophytes (coded EE) and epiphytes (coded E) separated from root (R), stem (S), and leaves (L). The endophytic bacterial isolate EES4 separated from the stem exhibited the most promising antimicrobial effect, with notable activity against *E. coli* ATCC 25,922, *S. aureus* ATCC 25,923, *C. albicans* ATCC 14,053, MDR *E. coli*, MDR *P. aeruginosa*, MDR *A. baumannii*, MDR *K. pneumoniae*, MDR *K. terrigena* (Table 2). Therefore, the EES4 isolate was selected for further evaluation.

**Table 2:** Antimicrobial preliminary screening of endophytic (EE) and epiphytic (E) bacterial isolates against standard and clinical pathogens

Isolate code	Standard strains			MDR clinical isolates				
	*C. albicans* ATCC 14,053	*S. aureus* ATCC 25,923	*E. coli* ATCC 25,922	*E. coli*	*P. aeruginosa*	*A. baumannii*	*K. pneumoniae*	*K. terrigena*
ER1	+	-	-	+	-	-	-	-
ER2	+	+	-	+	-	-	-	-
EER3	+	-	+	+	-	-	-	-
**EES4**	**+**	**+**	**+**	**+**	**+**	**+**	**+**	**+**
EES5	+	-	-	-	-	-	-	-
EL6	+	+	+	+	+	+	-	-
EER7	+	-	-	-	-	-	-	-
EES8	+	+	-	-	-	-	-	-
ER9	+	-	-	-	-	-	-	-
EER10	+	-	-	-	-	+	-	-
EEL11	+	+	-	-	-	-	-	-
ES12	+	+	+	+	+	+	-	-
ES13	-	-	-	-	-	-	-	-
ES14	+	+	-	-	-	-	N/A	N/A
ES15	+	+	-	-	-	-	N/A	N/A
EES16	-	-	-	-	-	-	-	-

+: Inhibits growth, -: no inhibition, N/A: not performed, EES4 showed activity against all the tested microbial strains and was selected for further experiments.

### Antimicrobial analysis of EES4 isolate extract

The ethyl acetate extract of isolate EES4 showed distinct zones of inhibition in comparison to the negative control as shown in Table 3. The agar well diffusion results were consistent with the perpendicular streak method results. The recovered EES4 extract showed broad antimicrobial activity against *E. coli* ATCC 25922, *S. aureus* ATCC 25923, *C. albicans* ATCC 14053, and MDR *P. aeruginosa* is delineated in Fig. S1.

**Table 3:** Antimicrobial activity of crude extract of the isolate EES4 against various standard strains and MDR pathogens

	Zone of inhibition (mm)					
	*C. albicans* ATCC 14,053	*S. aureus* ATCC 25,923	*E. col*i ATCC 25,923	MDR *P. aeruginosa*	MDR *A. baumannii*	MDR *K. pneumoniae*
Ethyl acetate extract	24	18	20	24	14	18

### Identification of EES4 isolate

The EES4 isolate was morphologically and genetically identified as *Bacillus licheniformis* isolate EES4. The complete genomic sequence (4125835 bp) was deposited in the NCBI GenBank with assigned accession number CP157373 (https://www.ncbi.nlm.nih.gov/nuccore/CP157373.1). It was found that this strain harbored a large plasmid (205548 bp) which was sequenced, assembled, annotated, and deposited under the accession number CP157373 (https://www.ncbi.nlm.nih.gov/nuccore/CP157374.1). The EES4 strain was deposited in the Culture Collection Ain Shams University (https://ccinfo.wdcm.org/collection/by_id/1186), a local culture collection under the accession code, *Bacillus licheniformis* strain CCASU-B18.

### AntiSMASH analysis

Aligning and analysis of the whole genome sequence of *Bacillus licheniformis* strain CCASU-B18 revealed a number of secondary antimicrobial metabolite biosynthetic gene clusters. The following metabolites are listed according to the percent of similarity of the respective biosynthetic gene clusters.

### Thermoactinoamide A

**Fig. 1:**
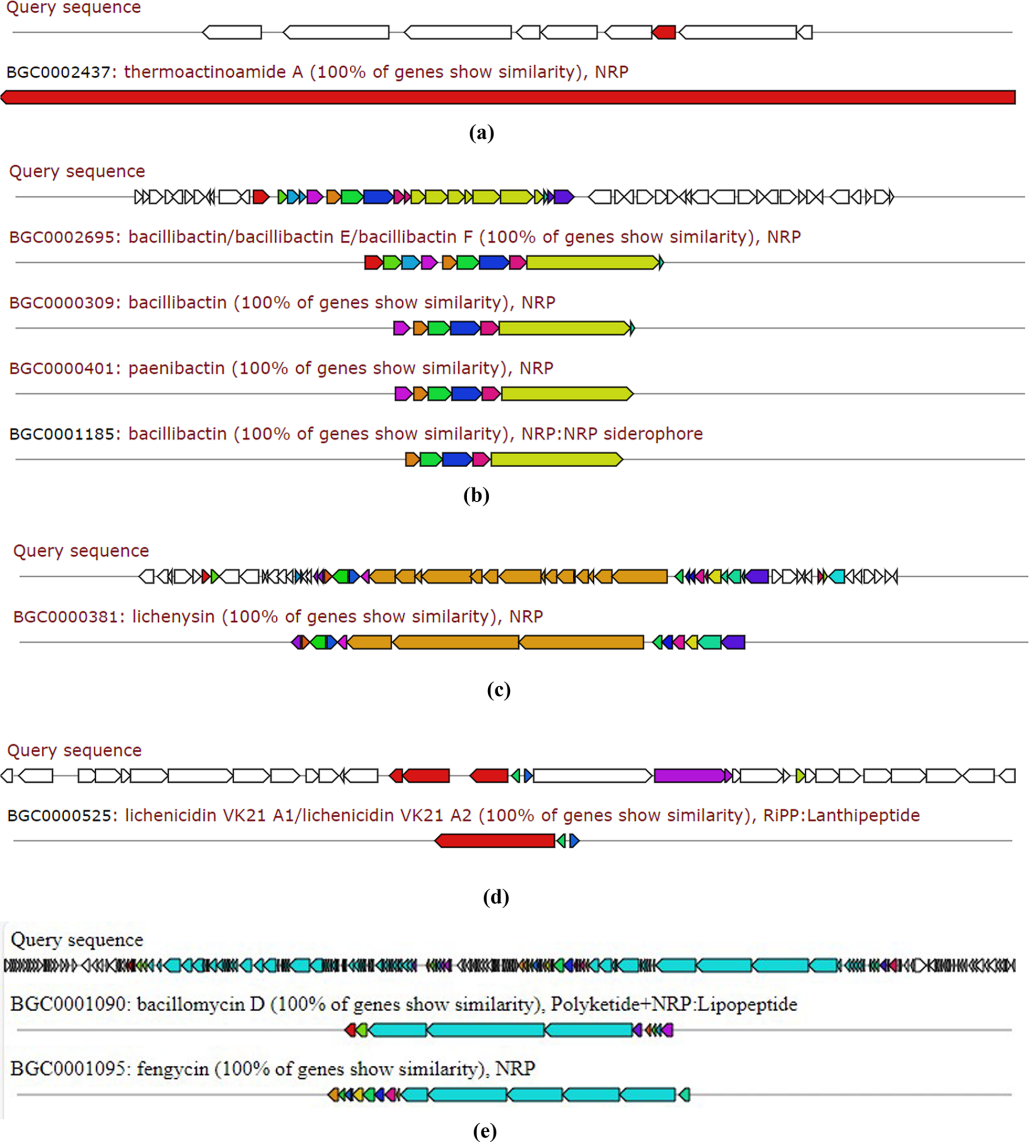
The biosynthetic gene clusters of *Bacillus licheniformis* strain CCASU-B18 identified using antiSMASH for the following metabolites: **a** thermoactinoamide A; **b** bacillibactin; **c** lichenysin; **d** lichenicidins and **e** fengycin and bacillomycin. The conserved biosynthetic gene(s) are colored

It is considered a newly discovered lipophilic cyclopeptide that has bactericidal activity against *S. aureus* (Teta et al. 2017). The biosynthetic gene cluster showed 100% similarity to *Thermoactinomyces* sp. AS95 (Fig. 1a).

### Bacillibactin/bacillibactin E/bacillibactin F

Bacillibactins are iron-scavenging molecules known as sidrephores that exhibit bactericidal activity against MDR bacteria (Chakraborty et al. 2022; Dimopoulou et al. 2021). The bacillibactins biosynthetic gene cluster of *Bacillus licheniformis* strain CCASU-B18 showed 100% similarity to that produced by *Bacillus* sp. WMMC1349. (Fig. 1b)

### Lichenysin

It is a valuable lipopeptide biosurfactant that is known for its anti-biofilm activity (Zammuto et al. 2023). Gene cluster showed 100% similarity to *Bacillus licheniformis* ATCC 14,580 (Fig. 1c).

### Lichenicidin VK21 A1/A2

Lichenicidins belong to a class of natural antibiotics called lantipeptides that contain lanthionine amino acid as part of their structure. Lantipeptides bactericidal effect is attributed to their ability to disrupt the cellular membrane and cell wall synthesis acquiring activity against MDR bacteria (Chakraborty et al. 2019). The lichenicidin gene cluster of *Bacillus licheniformis* strain CCASU-B18 showed 100% similarity to lichenicidin VK21 A1 /A2 producing gene cluster (Fig. 1d).

#### Antifungal metabolites fengycin and bacillomycin

Fengycin is an antifungal cyclic lipopeptide with activity against *C. albicans* (Rautela et al. 2014). *Bacillus licheniformis* CP157373- CP157374 showed 100% similarity to *Bacillus velezenensis* FZB42. Moreover, a bacillomycin gene cluster of *Bacillus licheniformis* strain CCASU-B18 showed 100% similarity to that of *B. amyloliquefaciens*. Bacillomycin is a potent antifungal metabolite produced by some bacilli such as *B. subtilis* and *B. amyloliquefaciens* as previously reported (Fig. 1e).

### LC-MS analysis

The LC/MS analysis of the obtained extract in both positive and negative ion modes resulted in the detection of multiple secondary metabolites that were consistent with the antiSMASH genomics analysis. The positive mode spectra peaks corresponded to the detection of the following protonated molecules thermoactinoamide B peak at *m/z* 702 (Fig. 2a), thermoactioamide H peak at *m/z* 686 (Fig. 2b), thermoactioamide J peak at *m/z* 680 (Fig. 2c), thermoactioamide K peak at *m/z* 652 (Fig. 2d), bacillibactin peak at *m/z* 883 ((Fig. 2e), fengycin peak at *m/z* 1464 (Fig. 2f). The negative mode spectra peaks corresponded to the detection of the following deprotonated molecules thermoactinoamide D peak at *m/z* 764 (Fig. 3a), thermoactioamide I peak at *m/z* 778 (Fig. 3b), thermoactioamide J peak at *m/z* 678 (Fig. 3c), bacillibactin peak at *m/z* 881 (Fig. 3d), lichenysin peak at *m/z* 1020 (Fig. 3e), fengycin peak at *m/z* 1462 (Fig. 3f).

**Fig. 2:**
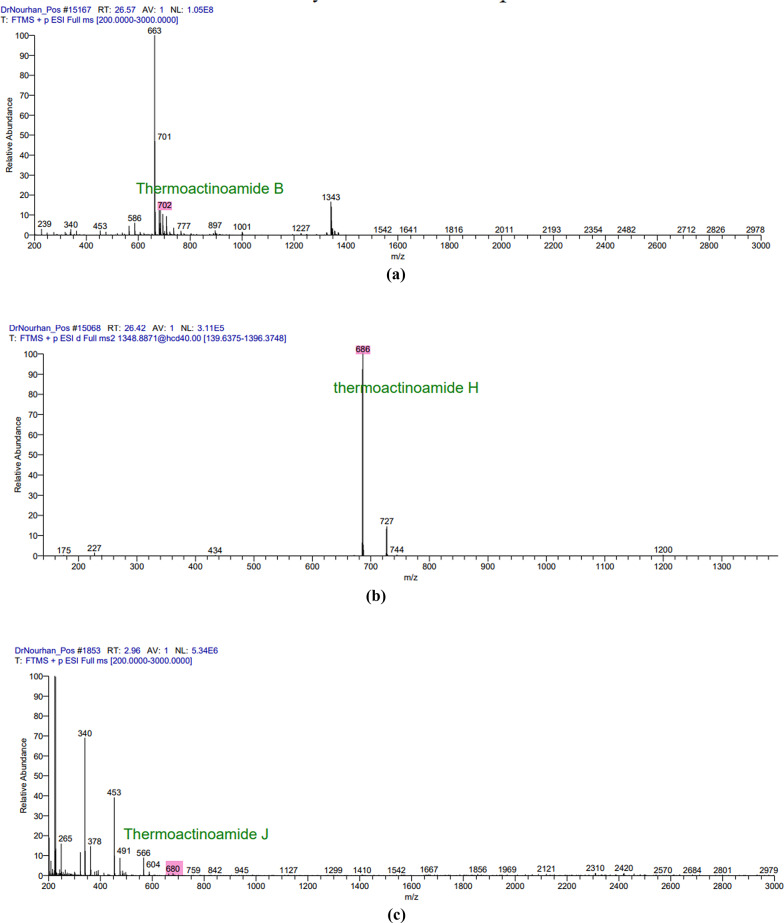
The positive mode spectra peaks of the LC/MS analysis for the following metabolites produced by *Bacillus licheniformis* strain CCASU-B18: **a** thermoactinoamide B; **b** thermoactinoamide H; **c** thermoactinoamide J; **d** thermoactinoamide K and **e** bacillibactin, **f** fengycin

**Fig. 2:**
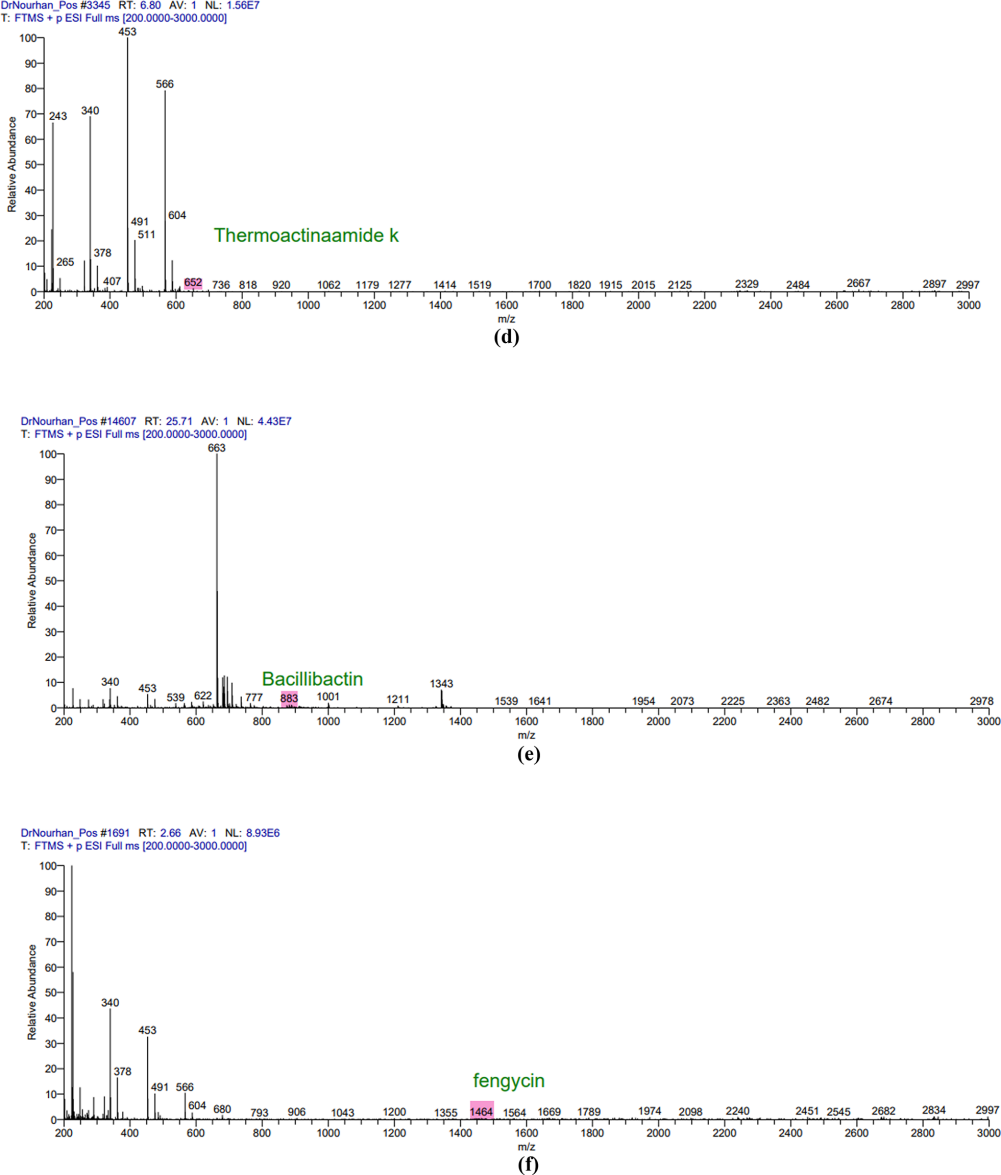
(continue)

**Fig. 3:**
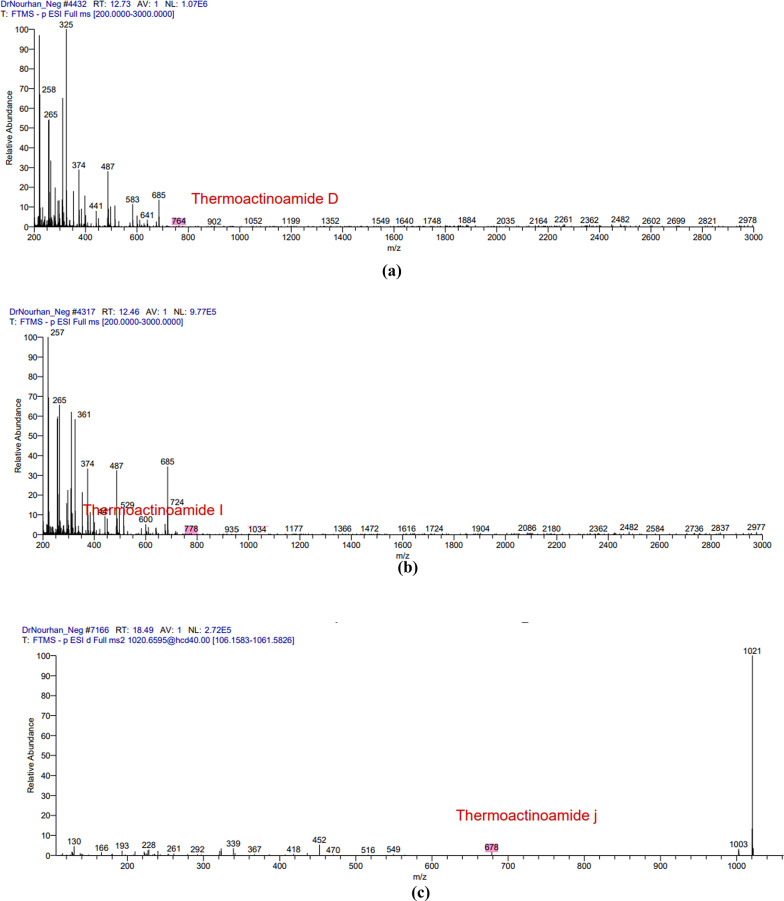
The negative mode spectra peaks of the LC/MS analysis for the following metabolites produced by *Bacillus licheniformis* strain CCASU-B18: **a** thermoactinoamide D; **b** thermoactinoamide I; **c** thermoactinoamide J; **d** bacillibactin **e** lichenysin, **f** fengycin

**Fig. 3:**
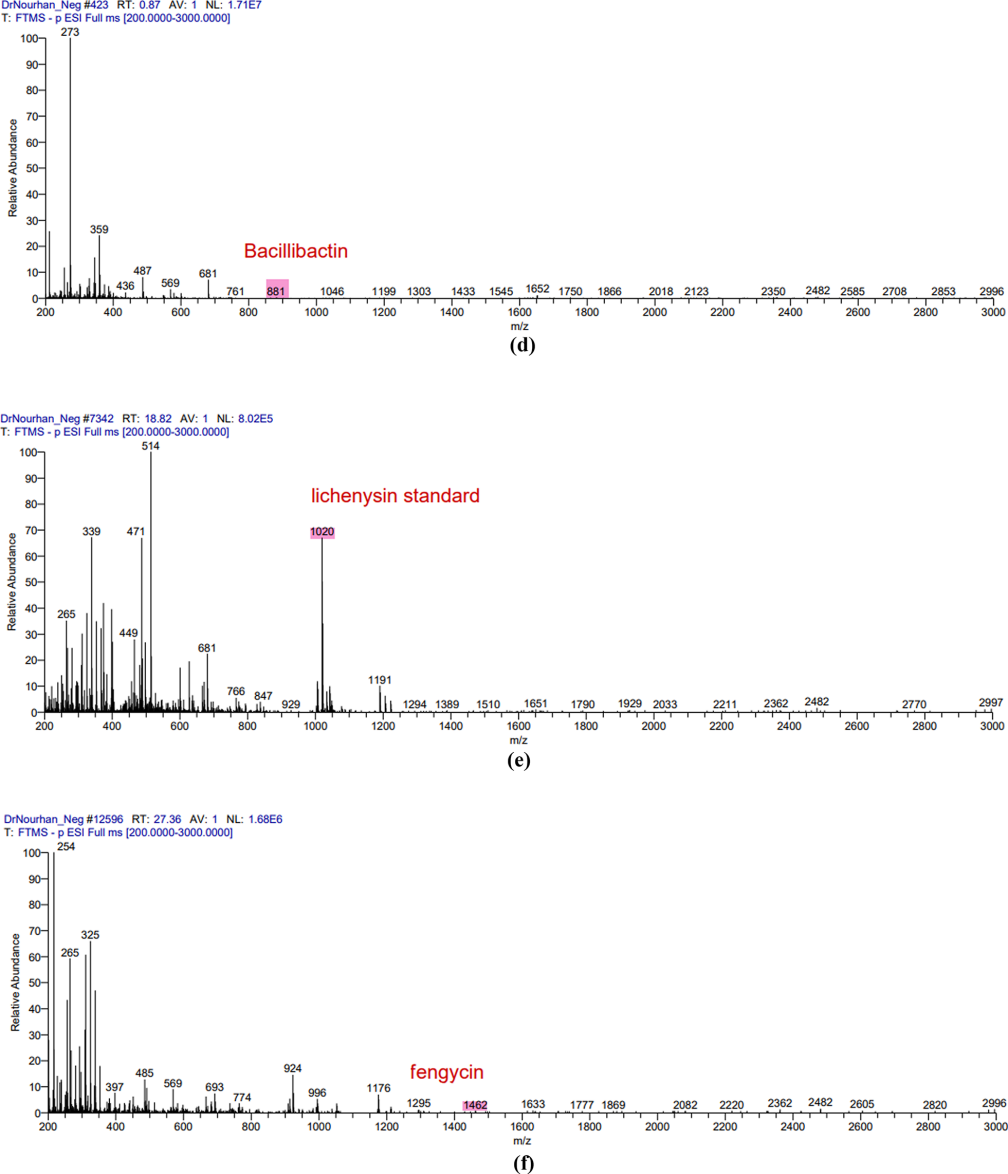
(continue)

## Discussion

The decline in the development of new antimicrobials has led to one of the most critical public health threats identified as antimicrobial resistance (Hutchings et al. 2019). Plants are hosts to an exceptional class of bacteria known as endophytes that have been a valuable source of various bioactive compounds (Martinez-Klimova et al. 2017). Endophytes assist plants greatly in overcoming stressful conditions therefore, the most promising endophytes have been associated with wild plants due to their ability to withstand extremely harsh conditions (Afzal et al. 2019). In the present study, a promising endophytic bacterium was isolated and identified through whole genomic sequencing as *B. licheniformis* with an accession number CP157373 on the NCBI GenBank database and deposited in the Culture Collection of Ain Shams University (CCASU) as *Bacillus licheniformis* strain CCASU-B18. This endophyte bacterium was isolated from the wild halophyte *Bassia scoparia* growing in Cairo, Egypt. To the best of our knowledge, this is the first report to investigate the antimicrobial capability of endophytic bacteria isolated from *Bassia scoparia*.

In this study, the tested isolate showed broad-spectrum antimicrobial activity against three standard microbial strains including, *C. albicans*,* S. aureus*,* E. coli*, as well as four MDR clinical pathogens including *E. coli*, *P. aeruginosa*,* (A) baumannii*, *K. pneumoniae* and *K. terrigena*. Our results are in accordance with previous studies that reported *(B) licheniformis* as a remarkable species which has been associated with various bioactive compounds. Such compounds included antibacterial, antifungal, antiviral, antibiofilm as well as anticancer agents (Shleeva et al. 2023; Alharbi et al. 2024). A study performed on *B. licheniformis* B116 strain isolated from soil showed antibacterial activity against *S. aureus*, *B. cereus*, *Listeria monocytogenes*, *Micrococcus luteus*, *E. coli*, and Salmonella enterica serovar (Guo et al. 2012). Another study on probiotic *B. licheniformis* MCC 2512 identified a new lantibiotic known as sublichenin that exhibited bactericidal effects against foodborne pathogens, enhancing the biosafety of the food system (Halami 2019). Moreover, *B. licheniformis* 09IDYM23 strain was reported to display antifungal activity as well as antibacterial activity due to the presence of new glycolipids. *B. licheniformis* 09IDYM23 had an effect on *S. aureus*, *B. subtilis*, *B. cereus*, Salmonella typhi serovar, *E. coli*, and *P. aeruginosa* in addition to antifungal activity against *(C) albicans* and other plant-related fungi (Tareq et al. 2015).

Following the confirmation of *B. licheniformis* strain CCASU-B18 antimicrobial activity, genomic analysis provided deeper insights into the biosynthetic potential of the isolate. A study conducted showed that antimicrobial metabolite genes constitute 5% of the whole *Bacillus* genus genome (Chen et al. 2007). Interestingly, in the present study, genomics analysis revealed the presence of six conserved (100% identity) antimicrobial biosynthetic gene clusters in the genome of *B. licheniformis* strain CCASU-B18. The first gene cluster showed 100% similarity to the biosynthetic gene cluster of thermoactinoamide A, a bioactive metabolite of *Thermoactinomyces sp.* AS95. This cyclic peptide showed bactericidal activity against *S. aureus* as well as antitumor activity (Teta et al. 2017; Della Sala et al. 2020). This gene cluster to the best of our knowledge, is the first time to be identified in a *Bacillus licheniformis* strain CCASU-B18 and the second time to be identified in the genus *Bacillus*, as stated by a very recent study that detected the presence of thermoactinoamide A gene cluster in the *B. subtilis* Tamang Srain genome (Prakash Tamang et al. 2024). Analyzing the LC/MS spectra, we detected the presence of thermoactinoamide B, D, H, I, J, and K in our aqueous extract, which are structural variants of themoactinoamide A (Della Sala et al. 2020). Therefore, the production of thermoactinoamide derivatives by *B. licheniformis* strain CCASU-B18 has been confirmed by genomic analysis and LC-MS analysis.

Moreover, antiSMASH analysis revealed a second biosynthetic gene cluster showing 100% similarity is lichenicidin VK21 A1/A2, these peptides were primarily isolated from *B. licheniformis* VK21 strain (Shenkarev et al. 2010). Lichenicidin VK21 A1/A2 are ribosomal peptides known as lantipeptides that target cell wall synthesis by targeting lipid II precursor. Lantibiotics are known to exhibit activity against Gram-positive bacteria such as methicillin-resistant *S. aureus* (MRSA) and vancomycin-resistant *enterococci* (VRE) (Breukink and de Kruijff 2006; Chakraborty et al. 2019). Previous studies are in line with our study as the aqueous extract of *B. licheniformis* strain CCASU-B18 showed bactericidal activity against *S. aureus* ATCC 25,923. However, the lichenicidin peptides were not identified in the LC/MS as lichenicidin VK21 A1 mass is 3249.51 Da and lichenicidin VK21 A2 mass is 3019.36 Da (Shenkarev et al. 2010). Whereas, the detection limit was 3000 *m/z*, preventing the identification of these molecules.

The bacillibactin class was the third biosynthetic gene cluster identified showing 100% similarity to the producing gene cluster. Bacillibactins are non-ribosomal peptides associated with various *Bacillus* strains that belong to a class known as sidrephores which cause low iron availability for other pathogens in the surrounding environment (Zhou et al. 2018). Bacillibactin E and bacillibactin F were identified in a recent study from a *Bacillus* strain linked to a marine sponge (Wu et al. 2021). To the best of our knowledge, this study provided the first evidence that *B. licheniformis* possesses the biosynthetic gene of bacillibactin E and bacillibactin F metabolites and they were both detected in the LC/MS spectra. Lichenysin is the fourth gene cluster identified showing 100% conservation. This gene has always been associated with *B.licheniformis* (Gudiña and Teixeira 2022*)*. Lichenysin is a unique, nontoxic cyclic lipopeptide biosurfactant that belongs to a class of biosurfactants known as surfactins. Microbial biosurfactants have significant industrial value not only for their high biodegradability but also for their antimicrobial and antibiofilm properties (Gudiña and Teixeira 2022). Our LC/MS results corresponded well with the antiSMASH analysis as lichenysin homologs molecular weights were detected from 993 to 1035 Da (Coronel-León et al. 2016; Zammuto et al. 2023). Culture media is key in the expression of lichenysin synthetase and in lichenysin homologs diversity (Gudiña and Teixeira 2022; Zammuto et al. 2023) hence, the culture media used in our study for fermentation is in line with lichenysin production.

Fengycin is another cyclic lipopeptide that is known for its fungicidal activity (Rautela et al. 2014). Although the fifth gene cluster shows 46% similarity with fengycin, however, the crude extract of *B. licheniformis* CP157373-CP157374 showed distinct activity against *Candida* spp. in addition to the detection of fengycin homologs in the LC/MS spectra 1450–1515 Da (Rautela et al. 2014). Fengycin homologues containing long acyl chains are known for their potent activity against C*andida* spp. (Rautela et al. 2014) and that can offer an understanding of our isolate’s significant anti-candidal activity. The antiSMASH analysis also revealed conservation of the biosynthetic gene cluster of bacillomycin D, however, it was not detected in the LC-MS spectra. A previous report confirmed the production of bacillomycin D by *B. amyloliquefaciens* (Gu et al. 2017)d *subtilis* (PEYPOUX et al. 1981)which has a potent antifungal activity, particularly against the plant-pathogenic fungus *Fusarium graminearum*. Wild halophytic plants, often considered troublesome in agriculture, can be the solution to the AMR crisis. Its resistance to biotic stress can be attributed to its ability to harbor unique endophytes. Endophytes, including *B. licheniformis*, produce exceptional secondary metabolites that possess different bioactivities of great potential that can be used in the discovery of new molecules (Chaudhary et al. 2022; Kamran et al. 2022). This is the first study to report themoactinoamide-A structural variants,, bacillibactins, lichenysins, lichenicidins, fengycin, and bacillomycin from *B. licheniformis* strain CCASU-B18. Future research could explore optimizing culture conditions to enhance the production of specific antimicrobial metabolites, as well as testing these compounds against a broader range of clinically relevant pathogens, particularly those conferring phenotypic resistance to existing antimicrobial agents used in clinical practice. In conclusion, a promising endophytic bacterium was isolated from *Bassia scoparia* plant and identified as *B. licheniformis* strain CCASU-B18. The respective endophytic bacterium exhibited broad-spectrum antibacterial activities against the standard and MDR clinical isolates, as well as antifungal activity against the standard *C. albicans* strain. The whole genome sequence, coupled with LC-MS analysis confirmed the presence of four major antimicrobial metabolites including thermoactinoamide A, bacillibactins, lichenysins, fengycin. However, lichenicidins, and bacillomycin were only confirmed by the identification of their biosynthetic gene clusters. Future research is highly recommended to optimize the culture conditions that will be employed to enhance the production of the respective antimicrobial metabolites, as well as testing these compounds against a broader range of MDR-resistant pathogens for future therapeutic potential. To the best of our knowledge, this is the first report to investigate the antimicrobial capability of endophytic bacteria isolated from *Bassia scoparia* plant.

## Electronic supplementary material

Below is the link to the electronic supplementary material.

Supplementary Material 1

## Data Availability

All data generated or analyzed during this study are included in this published article and supplementary file. The genomic DNA of the promising endophytic isolate(s) was analyzed, assembled, annotated and submitted into the NCBI GenBank database under the nucleotide accession code, CP157373 (https://www.ncbi.nlm.nih.gov/nuccore/CP157373) and the genome sequence files were submitted as BioSample ID, SAMN41059648 (https://www.ncbi.nlm.nih.gov/biosample/SAMN41059648/). The final assembled genome was submitted into the NCBI GenBank under accession code GCA_040047715.1 (https://www.ncbi.nlm.nih.gov/datasets/genome/GCA_040047715.1/).

## Abbreviations

AntiSMASH: Antibiotics and secondary metabolite analysis shell
AMR: Antimicrobial resistance
CCASU: Culture Collection Ain Shams University
MRSA: Methicillin-resistant *S. aureus*
DMSO: Dimethyl sulfoxide
HPLC: High-performance liquid chromatography
HPLC: High-performance liquid chromatography
LC-MS: Liquid chromatography mass spectroscopy
MDR: Multidrug-resistant
NCBI: National Center of Biotechnology Institute
VRE: Vancomycin-resistant enterococci
WHO: World Health Organization
