# Supplementary material for: Whole genome sequence and LC-Mass for identifying antimicrobial metabolites of Bacillus licheniformis endophyte
Source: AMB Express. 2024 Dec 20;14:139. doi: 10.1186/s13568-024-01789-y (PMC11662132; doi:10.1186/s13568-024-01789-y)
Supplement: Supplementary file 1 — Supplementary Material 1 [file 13568_2024_1789_MOESM1_ESM.docx]

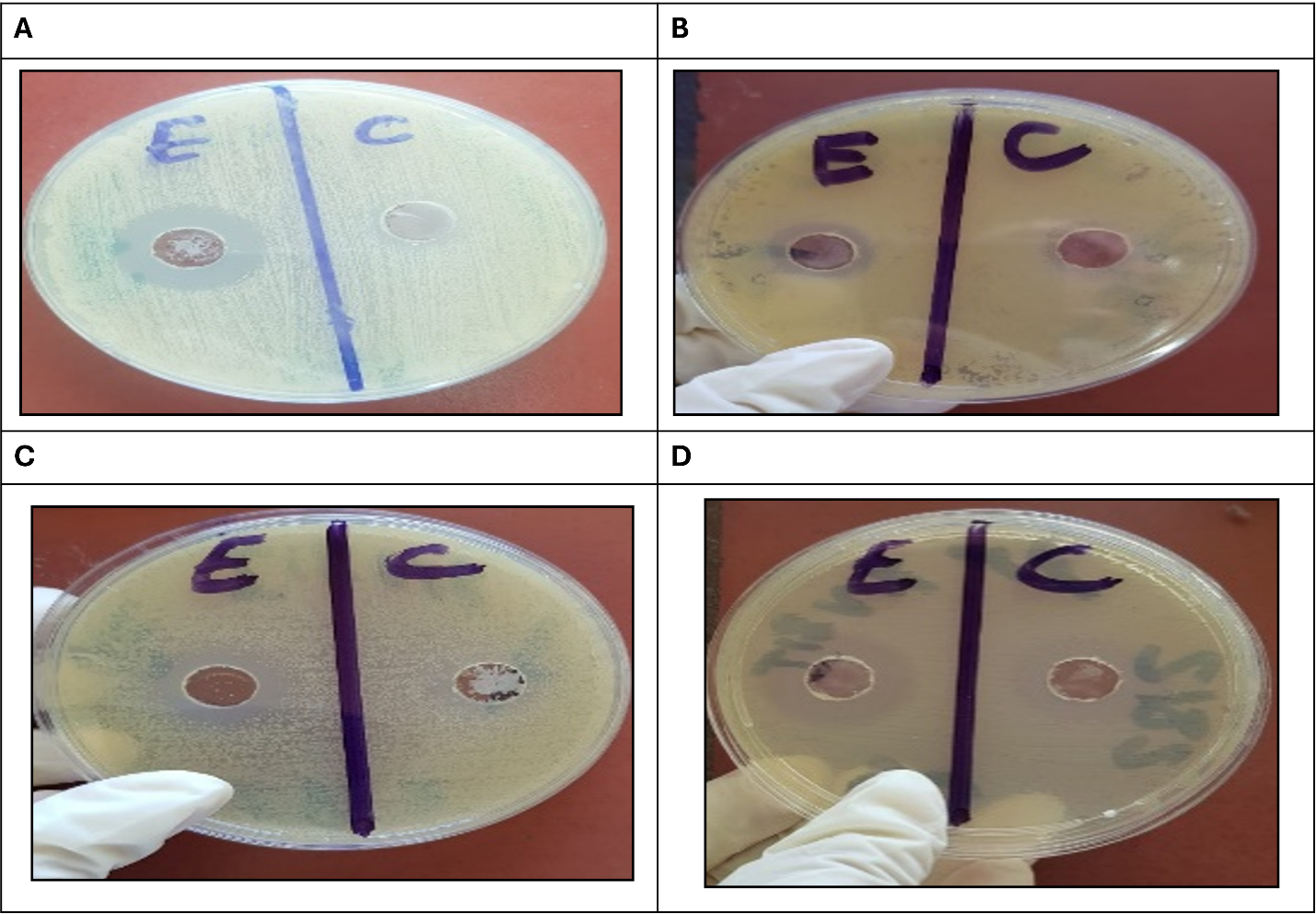


**Fig. S1.** Antimicrobial activity of EES4 organic extract against **A.** *C. albicans* ATCC 14053, **B.** *S. aureus* ATCC 25923, **C.** *E. coli* ATCC 25922, **D.** MDR *P. aeruginosa.* E, the tested endophyte extract, C, control (DMSO 60%)
